# Morphological and morphometric study of the scapulae of Korean wild deer

**DOI:** 10.1038/s41598-023-33730-x

**Published:** 2023-05-10

**Authors:** Myung-Cheon Kang, Jun Kwon, In-Shik Kim, Byung-Yong Park, Hyun-Jin Tae, Young-Jin Jang, Jeoungha Sim, Dongchoon Ahn

**Affiliations:** 1 Department of Companion Animal Health, Busan Kyungsang College, 170 Gobun-ro, Yeonje-gu, Busan 47583 Republic of Korea; 2 Department of Veterinary Anatomy, College of Veterinary Medicine and Institute of Animal Transplantation, Jeonbuk National University, 79 Gobong-ro, Iksan-si, Jeollabuk-do 54596 Republic of Korea; 3 Department of Nursing, College of Medical Science, Jeonju University, 303 Cheonjam-ro, Wansan-gu, Jeonju-si, Jeollabuk-do 55069 Republic of Korea

**Keywords:** Animal physiology, Skeleton

## Abstract

Korean water deer (*Hydropotes inermis argyropus*; Heude, 1884) and Siberian roe deer (*Capreolus pygargus*; Pallas, 1771) are Korean wild deer classified in the tribe Capreolini. *C. pygargus* in Korea were previously considered a single species; however, it was recently suggested that roe deer living on Jeju Island (Jeju roe deer; *Capreolus pygargus jejuensis*) is a distinct subspecies from roe deer living on the Korean peninsula (mainland roe deer; *Capreolus pygargus tianschanicus*) based on several studies demonstrating genetic and morphological features. In this study, we suggests that the scapular morphology and osteometric data can be used for interspecies discrmination between Korean wild deer. To compare the morphological characteristics of scapula among the three groups of deer, we analyzed the features and nine osteomorphological measurements of 31 *H. i. argyropus* (14 males and 17 females), 18 *C. p. jejuensis* (4 males and 14 females), and 23 *C. p. tianschanicus* (16 females and 7 males). The estimated ages of the deer were over 32–35 months. Data were analyzed by one-way repeated measures analysis of variance with post hoc Duncan test and discriminant functional analysis (DFA). *H. i. argyropus* and *C. p. tianschanicus* had the smallest and largest scapulae, respectively. The scapulae of the three Korean wild deer had a similar triangular shape, which was obscured by the tuber of the scapular spine, pointed acromion, broad infraspinous fossa, narrow supraspinous fossa, and partial ossification of scapular cartilage in older deer. *H. i. argyropus* had certain distinctive features, including a caudally pointed acromion, a notch between the supraglenoid tubercle and glenoid cavity (NBSG), a glenoid notch, and no sexual dimorphism, except for the longest dorsal length (Ld) and the scapular index (SI). *C. p. jejuensis* had a larger scapular index (SI) (61.74 ± 0.74%), compared with the SIs of *H. i. argyropus* and *C. p. tianschanicus*. The unique features of the scapula in *C. p. jejuensis* include its S-shaped cranial border. The *C. p. jejuensis* had a cranially pointed acromion, less frequent presence of glenoid notch and NBSG, short length of supraglenoid tubercle, and no sexual dimorphism. The *C. p. tianschanicus* had elevated cranial margin of the glenoid cavity, and frequent presence of glenoid notch and NBSG, similar to the *H. i. argyropus*. Similar to *C. p. jejuensis*, *C. p. tianschanicus* had a cranially pointed acromion. However, sexual dimorphism was observed in *C. p. tianschanicus*. DFA using osteometric data showed 97.22% of specimens were classified correctly into their species, meaning the osteometric parameters can be used for interspecies discrimination of Korean wild deer. Our findings indicate that the scapular morphologies of the three Korean wild deer have certain similarities and differences, suggesting that *C. p. jejuensis* are distinct from *C. p. tianschanicus*.

## Introduction

Skeletal characters can be induced by animals’ adaptation to their environments^1,2^. As a variety of factors, such as phylogeny, locomotor performance, external features, animal’s ecology, etc, are involved, the characters may provide enormous information of the animals. The pectoral girdle of tetrapoda consists of scapula, coracoid bone, and clavicle. As the main component of the shoulder girdle, the scapula is the attachment site for 13 shoulder girdle and forelimb muscles, and articulates with the humerus. The fact suggest that scapula morphology is one of the most important factors in locomotion performance^2–6^. In ruminants, the several features of their scapula has been studied^7–9^. In case of deer scapula, previous studies briefly described the morphologies of several species, such as the Black tailed deer (*Odocoileus hemionus columbianus*), European roe deer (*Capreolus capreolus*), fallow deer (*Dama dama*), pudu (*Pudu puda*), and moose (*Alces alces*)^10–14^. However, no study has investigated scapular of Korean wild deer. Recently, Castanos et al.^3^ suggested that the morphological features of the scapula, such as the minimum anteroposterior diameter of the scapular neck and the development of the articular process, can aid in interspecies discrimination.

Two wild deer species, Korean water deer (*Hydropotes inermis argyropus*) and Siberian roe deer (*Capreolus pygargus*), mainly inhabit South Korea. The number of *C. pygargus* has declined, whereas the *H. i. argyropus* have been thriving in terms of number and range^15^. The wild water deer and roe deer that inhabit South Korea belong to tribe Caprelini in subfamily Capreolinae^16,17^. *C. pygargus* was previously considered a single species^18–20^. However, several recent studies have proposed a new taxonomic designation, which classifies *C. pygargus* into two subspecies: mainland roe deer (*Capreolus pygargus tianschanicus*) and Jeju roe deer (*Capreolus pygargus jejuensis*). *C. p. tianschanicus* inhabits the Korea peninsula, whereas *C. p. jejuensis* inhabits Jeju Island^21–24^. Park et al.^21^ have described morphological differences in the skull and external forms of *H. i. argyropus*. Additionally, genomic differences between *C. p. tianschanicus* and *C. p. jejuensis* were recognized in partial sequences of mitochondrial DNA, supporting the new taxonomic designation^22–24^.

In this study, we analyzed the morphological features of scapulae in *H. i. argyropus* and roe deer. The animals used in this study were aged over 32-35 months. Left scapular morphology was analyzed using the methods described by Von den Driesch^25^.

## Results

### Scapular morphology

**Figure 1:**
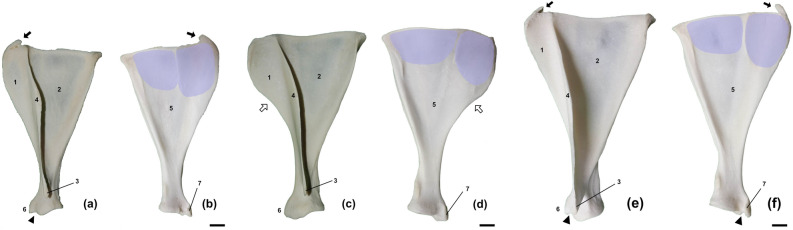
Scapula of Korean wild deer; (**a**, **b**) Korean water deer (*Hydropotes inermis argyropus*), (**c**, **d**) Jeju roe deer (*Capreolus pygargus jejuensis*, (**e**, **f**) mainland roe deer (*Capreolus pygargus tianschanicus*). Sinuous scapular spine with roughened border in the middle third and caudally pointed acromion is shown. A bony particle ossified in scapular cartilage in older animals aged > 4.5 years was fused with the scapular body at the cranial angle (arrow). S-shaped cranial border of *C. p. jejuensis* (white arrow). The notch between the glenoid cavity and the supraglenoid tubercle (NBSG) is shown (arrowhead). Lateral view (**a**, **c**, **e**) and medial view (**b**, **d**, **f**). Scale bar = 1 cm. Blue area; serrata surface. 1; supraspinous fossa, 2; infraspinous fossa, 3; acromion, 4; scapula spine, 5; subscapular fossa, 6; supraglenoid tubercle, 7; coracoid process.

**Figure 2:**
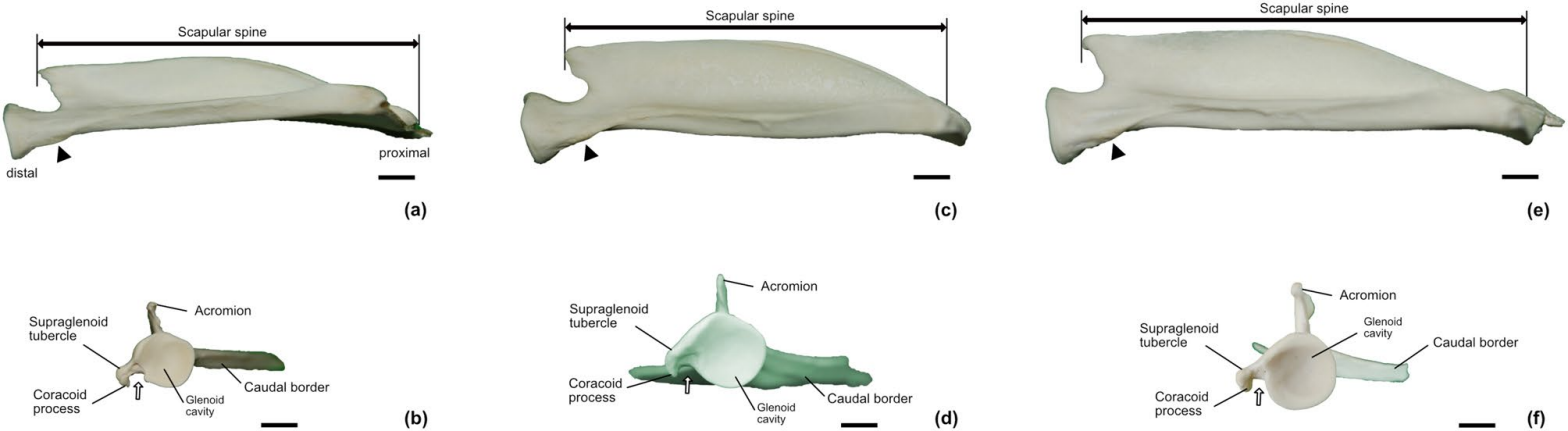
Scapula of Korean wild deer; (**a**, **b**) Korean water deer(*Hydropotes inermis argyropus*), (**c**, **d**) Jeju roe deer (*Capreolus pygargus jejuensis*, (**e**, **f**) mainland roe deer (*Capreolus pygargus tianschanicus*). The tuberosity of the scapular neck (arrowhead) and the glenoid notch (white arrow) are shown. The coracoid process extended medially from the tip of the supraglenoid tubercle. Caudal view (a,c,e) and ventral view (**b**, **d**, **f**). Scale bar = 1 cm.

Scapular spine extended from the dorsal border to the neck of scapula. Dividing lateral side of the scapula into supraspinous and infraspinous fossae, the scapular spine was noted at a level near one-fourth of the length of its dorsal edge. The size of the spine gradually increased until the scapular spine tuber which was located at the proximal third of the length of the spinous process (Fig. 1). The free margin of the scapular spine was sinuously curved; caudally at the proximal third of the scapular spine, distally at the scapular spine tuber, and then cranially at distal third of it. The scapular spine tuber was visible as a slightly thickened free margin, and the proximal region of the tuber was weaker than its distal region (Fig. 1). The direction of the acromion differed between *C. pygargus* and *H. i. argyropus*. The acromion of *H. i. argyropus* exhibited a caudal curvature, except in a single male deer, whereas the acromion of *C. pygargus* was cranially curved (Table 1). Additionally, the acromion was sharp in all deer, and its tip ended at the level of the scapular neck (Fig. 2). Among the three Korean wild deer, *H. i. argyropus* had the sharpest acromion. The supraspinous fossa was smaller than the infraspinous fossa. Both fossae were triangular, narrowed toward the scapular neck, and merged at the acromion level (Fig. 1).

The cranial border of the scapula (i.e., the line connecting the cranial angle to the ventral angle) was sharper than the caudal border. Additionally, the free margin was curved toward the scapular neck. In *H. i. argyropus*, the cranial border was oriented cranially from the cranial angle, then oriented obliquely toward the scapular neck at the level of the scapular spine tuber (Fig. 1). After formation of the scapular notch, the cranial border is oriented cranioventrally toward the supraglenoid tubercle (Fig. 1). The cranial border of *C. p. jejuensis* scapula had a well-developed curvature, similar to a gentle “S” shape (Fig. 1). It arose cranially from the cranial angle, was oriented along the scapular spine at an intermediate scapular height, and ended at the supraglenoid tubercle, making broad area of supraspinous fossa. In contrast, the cranial border of the Korean peninsula-dwelling *C. p. tianschanicus* was almost straight compared to other two deer (Fig. 1). The cranial border of the scapula began at the cranial angle and extended cranioventrally, which was similar to the cranial border of *H. i. argyropus*. Slightly dorsal to the level of the scapular spine tuber, it ran caudoventrally toward the scapular neck to form the scapular notch. Then, it continued cranioventrally to form the supraglenoid tubercle. The caudal border, located along the line connecting the caudal angle to the caudal margin of the glenoid fossa, was oriented straight toward the scapular neck; moreover, it was thicker than the cranial border. A tuberosity produced by the attachment of the teres minor muscle was present on the scapular neck. A nutrient foramen was located on the caudo-proximal margin of the tuberosity.

The subscapular fossa had a shallow concavity along the entire medial surface (costal surface). A round and low ridge was present at the caudal area of the fossa, forming the caudal scapular border (Fig. 1). The serrate surface extended from the cranial end to the caudal end of the medial surface, had a wide space at the cranial end, and became narrow caudally. The cranial and caudal serrate surfaces bordered the subscapular fossa along its lower ridge (Fig. 1).

Although scapular cartilage was lost during skeletal specimen preparation, there was clear evidence of its existence. Cartilage ossification is found in *C. pygargus* and *H. i. argyropus* aged> 4.5 years. The ossified cartilage is attached to the caudo-dorsal area of the cranial angle (Fig. 1). The cranial angle was round because the cranial border arose cranially, whereas the caudal angle was sharp and clear (Fig. 1). There were no significant differences in the ventral angle among the ruminant species. However, a notch between the glenoid cavity and supraglenoid tubercle (NBSG) was absent from 12 *C. p. jejuensis* (12/18; 66.7%). The coracoid process was clearly observed in all species (Table 1, Fig. 2). The rate of glenoid notch appearance differed among species. The glenoid notch was commonly observed in *H. i. argyropus* (71%) and *C. p. tianschanicus* (78.3%), whereas it was absent from *C. p. jejuensis* (44.4%) (Table 2, Fig. 2). No synovial fossa was present in the deer.

### Osteometric data

The osteometric data are presented in Table 3. With the exception of Ld (longest dorsal length), the osteometric data significantly differed among species.The osteometric parameters include HS (height), DHA (diagonal height), Ld, SLC (shortest length of the scapular neck), GLP (longest length of articular process), LG (length of the glenoid cavity), BG (breadth of the glenoid cavity). The Ld was shorter in *H. i. argyropus* than in the other species, whereas it was similar between *C. p. tianschanicus* and *C. p. jejuensis* (Table 3). The LTS (length of supraglenoid tubercle) was significantly shorter in *C. p. jejuensis* than in the other species. The SIs (scapula index) were 61.7%, 56.5%, and 56.1% in *C. p. jejuensis*, *C. p. tianschanicus*, and *H. i. argyropus*, respectively.

The result of discriminant functional analysis (DFA) showed that 97.2% of Korean wild deer were clustered accurately within each species (Fig. 4). Among 72 specimens, 2 *C. p. jejuensis* were misclassified as *C. p. tianschanicus*. About 91.6% specimens were assigned correctly in cross-validation procedure (*p*< 0.01) (supplementary data). The discriminant function 1 completely seperated *H. i. argyropus* and *C. p. tianschanicus*, but *C. p. jejuensis* overlapped with the other groups. On the other hand, the discriminant function 2 separated *C. p. jejuensis* from *C. p. tianschanicus* and *H. i. argyropus* groups. Among the predictors loadings on the discriminant function 1, HS, SLC, DHA, and BG were predictors which mainly contributed to seperating *H. i. argyropus* and *C. p. tianschanicus*, and DHA and BG contributed in it. The variables, HS, DHA, Ld, and LG, largely involved in discriminant function 2 which seperated *C. p. jejuensis* from the other species.

We compared the measurements between males and females (Table 3). In *C. p. tianschanicus*, the HS, Ld, SLC, GLP, LG, and BG values differed between sexes (HS, Ld, SLC, GLP, LG; p < 0.05, BG; p < 0.01). In *H. i. argyropus*, only Ld and SI differed between sexes (SI; p < 0.05, Ld; p < 0.01). No significant differences were observed between sexes in *C. p. jejuensis*.

## Discussion

Previous studies showed the scapulae can provide distinguishing indices between species^3,26,27^. The indices include not only the osteometric features but also morphologies including presence of several notches, and direction of acromion. Here, we present scapular indices of *H. i. argyropus*, *C. p. tianschanicus*, and *C. p. jejuensis*. The differences between species and subspecies suggest the diagnostic functions of the scapular indices.

The HS and Ld measurements of Korean wild deer showed significant differences in the order of *H. i. argyropus*, *C. p. jejuensis*, and *C. p. tianschanicus* (p<0.01). This result is consistent with Park et al.’s^21^ findings of body size differences among these species. Body size can be influenced by various habitat conditions, and the variation in HS and Ld, which directly reflects overall scapular size, appears to be the consequence of adaptation to environmental conditions. The ratio of HS and Ld (SI) was higher in *C. p. jejuensis* compared to the other deer species ($p<$0.01). This means that the scapula of *C. p. jejuensis* is relatively wider compared to other scapulae. Matsuo et al.^26^ found that ungulates living in mountainous areas have wider scapulae than those living in open habitats. This finding correlates with the habitat preference of *C. p. jejuensis*, which is mainly found in oreums (small volcanic cones or hills that are formed by the accumulation of volcanic material around a vent) and Halla Mountain on Jeju Island^28^. Therefore, the distinct SI value of *C. p. jejuensis* seems to reflect its adaptation to the environmental conditions of Jeju Island.

The supra/infraspinous fossa is the attachment site of the supra/infraspinatus muscle and plays a significant role in determining muscle features^1,2,26^. Because the morphology of these fossae mainly depends on the position of scapular spine and the cranial border of the scapula, *C. p. jejuensis* which has a gentle “S” shape of cranial border of scapula possesses relatively larger supraspinous fossa compared to the other two species that has the straight cranial border of scapula. Conversely, the infraspinous fossa of *C. p. tianschanicus* is relatively larger than that of *H. i. argyropus* and *C. p. jejuensis*. The differences of morphology of supra/infraspinous fossae are associated with the supra/infraspinatus muscle features and the their locomotion capacity^26^. These morphological features may be induced by adaptations to their mountainous habitats and locomotion performance on sloped areas after considering the function of supra/infraspinatus muscle and the deer’s habitat.

The scapular spines of Korean wild deer share several features with other ruminants. A weak scapular spine tuber, a thickened free margin of scapular spine were observed. The scapular spine arises from the dorsal border and curves caudally at the scapular spine tuber, which is similar to the morphologies in other ruminant species (except gorals)^8–10,13^. On the other hands, we found that the scapular spine slightly differed among species at the acromion level: the scapular spine was slightly inclined caudally in *H. i. argyropus*, whereas it was inclined cranially in *C. p. jejuensis* and *C. p. tianschanicus* (66.7% and 69.6%, respectively) (Table 1). Hildebrand^10^ found that scapular spine straightness could not be used to distinguish among species. However, previous studies^29–31^ showed that mechanical stress on the skeleton causes skeletal development during prenatal and postnatal periods. Therefore, the different inclines of scapular spine can be affected by animal’s habitual behaviors and adjusting to their habitats. The cranial acromion curve may result from the strong actions of supraspinatus and omotransverse muscles and the caudal curve could indicate that the caudal muscles attached to the scapula (i.e., thoracic region of the trapezius muscle, shoulder region of the deltoid muscle, and the infraspinatus muscle) are relatively larger in these taxa.

Although the development of scapular cartilage and its ossification with age in ruminant animals have been well-documented^7,8^, there is a lack of studies on the location and timing of this ossification. For instance, in horses, scapular cartilage appears after birth and fuses to the scapula after 3 years^8^. Wißdorf and Butendieck (1988)^13^ identified the initial structure that becomes ossified in adult pudu deer shoulder cartilage; the structure also becomes attached to the cranial angle. In this study, bone fragments were observed in scapular cartilage mainly in deer aged over 4.5 years, suggesting that scapular cartilage ossifies with age in *H. i. argyropus*, *C. p. jejuensis*, and *C. p. tianschanicus*. The bone fragments in scapular cartilage exist at the cranial angle and near the caudal angle or dorsal border, with fragments near the cranial angle fusing with the scapula earlier than others. However, further research is required to determine the number of bone formation centers (ossification centers) in scapular cartilage, the timing of their appearance, and the timing of their fusion with the scapula.

The tuberosity associated with the attachment of the teres minor muscle, was clearly observed on caudal border of scapula in *H. i. argyropus*, *C. p. jejuensis*, and *C. p. tianschanicus*. Although this structure is evident in Bovidae animals, it is not frequently mentioned in books^7–9^, International Committee on Veterinary Gross Anomorphic Nomenclature^32,33^, or previous studies^10,13,34–36^. However, Nickel et al. (1986)^9^ meontioned ridges on the caudal border close to the glenoid cavity in domestic ruminants, whereas Peters and Brink^37^ observed this structure in two members of the Bovidae (springbok and grey rhebok).

In most animals, supraglenoid tubercle is connected to the glenoid cavity without a clear boundary although there is another ossification center that forms the tubercle and the cranial portion of glenoid cavity. However, in the present study, we noted a distinctive difference in scapulae of *H. i. argyropus*, and *C. p. tianschanicus* in terms of the presence of a clear boundary between the glenoid cavity and supraglenoid tubercle. The clear boundary formed the NBSG as shown in Table 2. Although these differences may not necessarily lead to differences in muscle attachment, they may be useful for species identification.

The glenoid cavity is the portion of the scapula that articulates with the humerus, and in ruminants, its margin is generally circular or elliptical, according to several studies^3,8,9,37^. While synovial fossa is present in some species like cattle, sheep, and goral, it is not found in others, such as pudu, fallow deer^13^, fossils of *Rangifer tarandus*, and *Cervus elaphus*^3^. In the current study, synovial fossa was not noted in scapulae of Korean wild deer.

Glenoid notch is a distinct structure which usually referred in horse osteology^8^. In ruminants, the notch is present depending on species such as goral^38^, buffalo^35^, and pudu deer^13^. We observed the glenoid notch in Korean wild deer. The rate of glenoid notch presence was lower in *C. p. jejuensis* than in *H. i. argyropus* and *C. p. tianschanicus* (Table 2).

To our knowledge, this is the first study to evaluate scapular measurements in *H. i. argyropus* and roe deer. We found that the measurements-except Ld, LTS, and SI-were largest in *C. p. tianschanicus*, followed by *C. p. jejuensis* and *H. i. argyropus*. The Ld values were similar in *C. p. jejuensis* and *C. p. tianschanicus*, whereas the Ld of the *H. i. argyropus* was smaller. However, the LTS values of *H. i. argyropus* and *C. p. tianschanicus* were similar, whereas the LTS of the *C. p. jejuensis* was smaller. Consistent with the Ld results, the SIs of *H. i. argyropus* and *C. p. tianschanicus* were similar (both 56%), whereas the SI of *C. p. jejuensis* was bigger than other two deer (62%). Thus, the scapula was lower triangular shape in *C. p. jejuensis* than in *H. i. argyropus* or *C. p. tianschanicus*. This is because the Ld did not differ between *C. p. tianschanicus* and *C. p. jejuensis*, whereas the HS differed between species. This difference may be related to the smaller body size and shorter leg length of *C. p. jejuensis*, compared with *C. p. tianschanicus*^21^. Although SIs are difficult to compare among species because these values were not consistently assessed in previous studies, Getty ^8^ remarked an SI of 60% in cattle; Hildebrand (1955)^10^ found SIs of 62.5% in white-tailed deer, 69.0% in sheep, and 70.0% in goats; and Singh and Johari (1974)^36^ found SIs of 45% in cattle and 56% in buffalo. The scapulae of *H. i. argyropus* and *C. p. tianschanicus* are similar to the scapulae of buffalo, whereas the scapulae of *C. p. jejuensis* are similar to the scapulae of white-tailed deer.

Sex differences were observed in the Ld and SI of *H. i. argyropus* and in the HS, Ld, SLC, GLP, LG, and BG of *C. p. tianschanicus*; however, no sex differences were observed in any measurements of *C. p. jejuensis*. The SI of the male *H. i. argyropus* is larger than the female because of a disparity in Ld scale. There is no sex difference in the SI of the *C. p. tianschanicus*. Notably, other measurments except for SI, LTS, and DHA of *C. p. tianschanicus* showed sexual dimorphism, but *C. p. jejuensis* did not. While deer exhibit sexual dimorphism in antlers and canines, there is no any study reporting sexual dimorphism in scapula. However, previous studies^18–20^ have revealed scapular sexual dimorphism in some animal species, and there were different features between species. Therefore, the presence of sexual dimorphism can be used for interspecies distinction between *C. p. jejuensis* and *C. p. tianschanicus*.

In conclusion, we analyzed scapular sizes and shapes in *H. i. argyropus*, *C. p. jejuensis*, and *C. p. tianschanicus*, and found differences among the three deer. Morphologically, *C. p. jejuensis* demonstrated various differences compared to *C. p. tianschanicus* including the shape of the cranial border, the rate of glenoid notch presence and the presence of the NBSG. *H. i. argyropus* showed different acromion direction. In all osteometric data except LTS and SI, *C. p. tianschanicus* showed largest followed by *C. p. jejuensis* and *H. i. argyropus*. In SI, the Jeju roe deer was larger and the other two were similar. Sexual dimorphism was noted in SI and Ld of *H. i. argyropus* and 6 parameters (HS, Ld, SLC, GLP, LG, and BG) of *C. p. tianschanicus*, but not in *C. p. jejuensis*.These findings may provide an anatomical basis for the categorization of *C. p. jejuensis* and *C. p. tianschanicus* into subspecies.

## Materials and methods

### Sample collection

Animal carcasses were collected during 2004–2016 and donated by wildlife rescue centers, a veterinary service laboratory, and Jeju National University. Because water deer and roe deer in Korean peninsula are born between May and July, the date of birth of each deer was estimated^18^. Tooth eruption and secondary ossification centers in the vertebral body and epiphysis of limb bones were analyzed to determine the age of the deer^39,40^; animals aged over 32–35 months were used in this study. The study included 31 Korean water deer (*H. i. argyropus*; 16 females and 15 males), 18 Jeju roe deer (*C. p. jejuensis*; 14 females and 4 males), and 23 mainland roe deer (*C. p. tianschanicus*; 16 females and 7 males).

### Morphometric data collection

**Figure 3:**
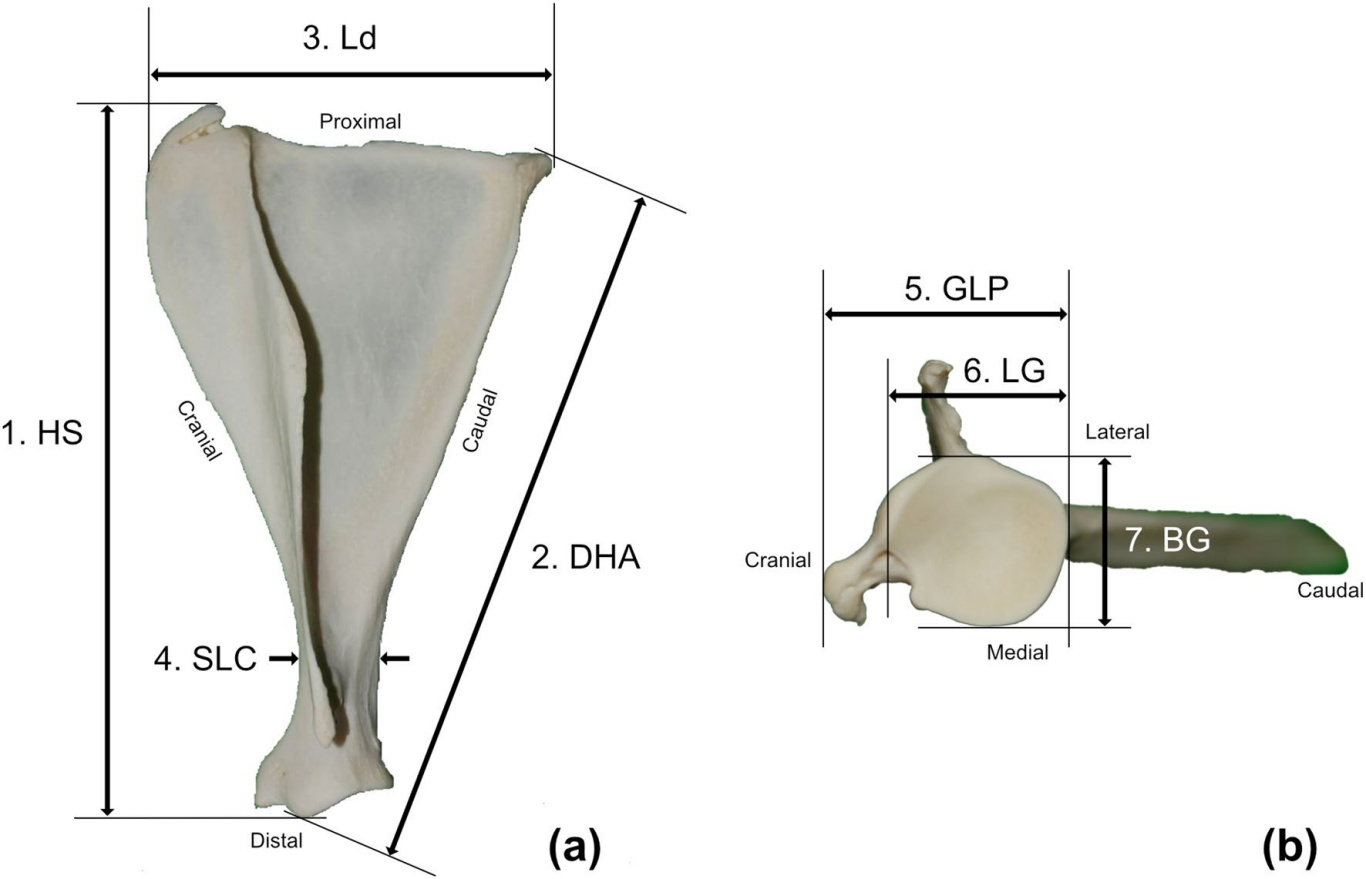
Scapular measurements in three Korean wild deer: Korean water deer (*Hydropotes inermis argyropus*), and Siberian roe deer that inhabit Jeju Island (Jeju roe deer; *Capreolus pygargus jejuensis*) and the Korean peninsula (mainland roe deer; *Capreolus pygargus tianschanicus*). 7(**a**); lateral view and 7(**b**); ventral view of the scapula in *H. i. argyropus*. HS: height, DHA: diagonal height, Ld: longest dorsal length, SLC: shortest length of the collum scapulae, GLP: longest length of processus articularis, LG: length of the glenoid cavity, BG: breadth of the glenoid cavity.

Figure 3 shows the scapular position during evaluation of morphological features. The sides of the supraspinous and infraspinous fossae were considered cranial and caudal, respectively. Morphological data were collected in accordance with the method established by von Den Driesch^25^. We measured the maximum scapular length along the spine (i.e., height of scapula [HS]), maximum length from the most caudal point of the scapula to the ventral angle (i.e., diagonal height [DHA]), maximum length from the cranial angle to the caudal angle (i.e., Ld), shortest length of the scapular neck (SLC), maximum length of ventral angle (longest length of the articular process [GLP]), length of the glenoid cavity (LG) measured parallel to the GLP, maximum breadth of the glenoid angle (i.e., breadth of the glenoid cavity [BG]), the difference between the longest length of the articular process and the length of the glenoid cavity (LG; i.e., length of the supraglenoid tubercle [LTS]), and proportion of Ld to HS (i.e., scapular index [SI]; calculated as [Ld/HS] $\times$ 100).

### Statistical analysis

Data were analyzed using SAS software (version 9.02; SAS Institute, Inc., Cary, NC, USA) and analysis of variance with Tukey’s post hoc test. p < 0.05 was considered indicative of statistical significance. The analyzed results between parameters were provided in supplementary data. Discriminant functional analysis was performed using caret version 6.0-93, ggplot2 version 3.4.1 and MASS version 7.3-58.1 package in R software version 4.2.2. Among the scapula morphology data, only measurements were used in the analysis; HS, DHA, Ld, SLC, GLP, LG, BG, and LTS. The data-set was standardised and used to establish classification of wild deer species. To verify the robustness of the classification, leave-one-out cross validation was used in R software.

**Figure 4:**
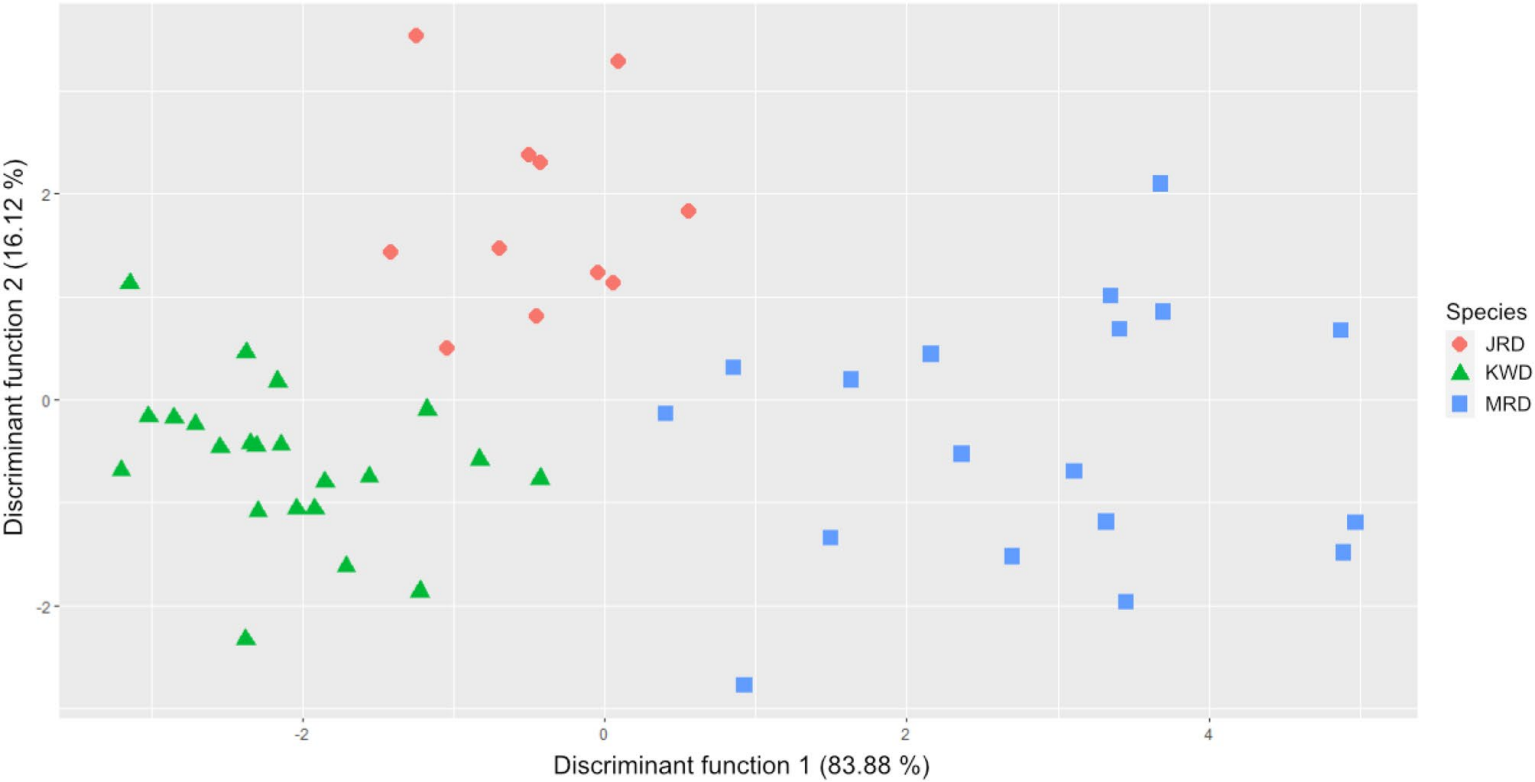
Discriminant functional anylsis using scapula osteometric parameters. A dot represents each sample and different colors represent different species, blue; MRD, mainland roe deer (*Capreolus pygargus tianschanicus*), red; JRD, Jeju roe deer (*Capreolus pygargus jejuensis*), green; KWD, Korean water deer (*Hydropotes inermis argyropus*).

**Table 1:** Direction of acromion in Korean wild deer. *; Two deer had different acromion direction of scapular spine between left and right scapula.

Direction of acromion	*H. i. argyropus*	*C. p. jejuensis*	*C. p. tianschanicus*
Cranial	1 (3.2%)	12 (66.7%)	16 (69.6%)*
Ventral	–	2(11.1%)	3 (13%)
Caudal	30 (96.7%)	4 (22.2%)	4 (17.4%)*
Total	31 (100%)	18 (100%)	23 (100%)

**Table 2:** Appearance of the glenoid notch and the notch between glenoid cavity and supraglenoid tubercle (NBSG) in Korean wild deer.

		*H. i. argyropus*	*C. p. jejuensis*	*C. p. tianschanicus*
Glenoid notch	Present	22 (71.0%)	8 (44.4%)	18 (78.3%)
	Absent	9 (29.0%)	11 (55.6%)	5 (21.7%)
	Total	31 (100%)	18 (100%)	23 (100%)
NBSG	Present	31 (100%)	6 (33.3%)	23 (100%)
	Absent	–	12 (66.7%)	–
	Total	31 (100%)	18 (100%)	23 (100%)

**Table 3:** Measurements of the parameters of scapula in 3 Korean wild deer (Mean± S.E.M, mm).

Parameters	*H. i. argyropus*			*C. p. jejuensis*			*C. p. tianschanicus*		
	All data	Male	Female	All data	Male	Female	All data	Male	Female
HS	121.51 ± 0.77	122.76 ± 1.18	120.32 ± 0.93	128.05 ± 1.30	131.31 ± 2.07	127.13 ± 1.51	145.60 ± 1.39	156.06 ± 2.37	143.65 ± 1.49
DHA	124.64 ± 0.86	125.99 ± 1.27	123.35 ± 1.12	133.68 ± 1.28	136.40 ± 2.19	132.94 ± 1.50	148.46 ± 1.36	151.96 ± 1.76	146.92 ± 1.69
Ld	68.15 ± 0.73	70.08 ± 1.14	66.40 ± 0.71	78.99 ± 0.95	83.40 ± 2.03	77.74 ± 0.84	82.41 ± 1.30	87.51 ± 2.18	80.18 ± 1.29
SLC	12.98 ± 0.16	13.19 ± 0.15	12.70 ± 0.25	15.28 ± 0.19	15.70 ± 0.23	15.11 ± 0.23	16.56 ± 0.31	17.57 ± 0.42	16.12 ± 0.37
GLP	24.77 ± 0.18	25.02 ± 0.27	24.53 ± 0.25	26.42 ± 0.23	26.68 ± 0.15	26.35 ± 0.29	28.20 ± 0.43	29.86 ± 0.53	28.08 ± 0.35
LG	18.55 ± 0.13	18.73 ± 0.19	18.36 ± 0.18	20.58 ± 0.30	21.22 ± 0.15	20.21 ± 0.37	21.91 ± 0.33	22.61 ± 0.63	21.61 ± 0.38
BG	17.77 ± 0.17	17.84 ± 0.28	17.67 ± 0.21	19.48 ± 0.22	19.84 ± 0.28	19.29 ± 0.29	21.03 ± 0.31	22.55 ± 0.35	20.36 ± 0.29
LTS	6.16 ± 0.14	6.18 ± 0.19	6.14 ± 0.20	5.55 ± 0.22	4.76 ± 0.62	5.78 ± 0.20	6.37 ± 0.19	6.57 ± 0.34	6.29 ± 0.24
SI(%)	56.11 ± 0.47	57.09 ± 0.75	55.20 ± 0.51	61.74 ± 0.74	63.55 ± 1.71	61.23 ± 0.80	56.62 ± 0.76	55.37 ± 1.12	55.86 ± 0.87

HS: height, DHA: diagonal height, Ld: longest dorsal length, SLC: shortest length of the collum scapulae, GLP: longest length of processus articularis, LG: length of the glenoid cavity, BG: breadth of the glenoid cavity, LTS: length of the tuberculum supraglenoidale, SI: scapular index; calculated as [Ld/HS] $\times$ 100%.

For data citations of datasets uploaded to e.g. *figshare*, please use the SPSVERBc1 option in the bib entry to specify the platform and the link, as in the SPSVERBc2 example in the sample bibliography file.

## Supplementary Information

Supplementary Information.

## Data Availability

All data generated or analysed during this study are included in this published article and its supplementary file.
